# Program on high value cost-conscious education in intensive care: *Educational program on prediction of outcome and cost awareness on Intensive Care admission*

**DOI:** 10.1186/s12909-020-02100-w

**Published:** 2020-06-08

**Authors:** L. van Wagenberg, C. J. P. Beurskens, I. Stegeman, M. C. A. Müller

**Affiliations:** 1 Department of Intensive Care Medicine, Amsterdam UMC, University of Amsterdam, Meibergdreef 9, 1105 AZ Amsterdam, the Netherlands; 2 Department of Paediatrics, Paediatric Intensive Care, Wilhelmina Children’s Hospital, University Medical Centre Utrecht, Lundlaan 6, 3584 EC Utrecht, the Netherlands; 3 Department of Otorhinolaryngology and Head & Neck Surgery, University Medical Centre Utrecht, Heidelberglaan 100, 3584 CX Utrecht, The Netherlands; 4 Brain Centre Rudolf Magnus, University Medical Center Utrecht, Heidelberglaan 100, 3584 CX Utrecht, the Netherlands; 5 Department of Clinical Epidemiology, Biostatistics and Bioinformatics, Amsterdam UMC, University of Amsterdam, Meibergdreef 9, 1105 AZ Amsterdam, the Netherlands

**Keywords:** High-value cost-conscious care, Post-graduate education, Curriculum development, Evaluation of education

## Abstract

**Background:**

Intensive Care (ICU) involves extended and long lasting support of vital functions and organs. However, current training programs of ICU residents mainly focus on extended support of vital functions and barely involve training on cost-awareness and outcome. We incorporated an educational program on high-value cost-conscious care for residents and fellows on our ICU and measured the effect of education.

**Methods:**

A cohort study with factorial survey design, in which ICU residents and fellows were asked to evaluate clinical vignettes, was performed on the mixed surgical-medical ICU of the Amsterdam University Medical Centre. Residents were offered an educational program focusing on outcome and costs of ICU care. Before and after the program they filled out a questionnaire, which consisted of 23 vignettes, in which known predictors of outcome of community acquired pneumonia (CAP), pancreatitis, acute respiratory distress syndrome (ARDS) and cardiac arrest were presented, together with varying patient factors (age, body mass index (BMI), acute kidney failure (AKI) and haemato-oncological malignancy). Participants were asked to either admit the patient or estimate mortality.

**Results:**

BMI, haemato-oncological malignancy and severity of pancreatitis were discriminative for admission to ICU in clinical vignettes on pancreatitis and CAP. After education, only severity of pancreatitis was judged as discriminative. Before the intervention only location of cardiac arrest (in- vs out of hospital) was distinctive for mortality, afterwards this changed to presence of haemato-oncological malignancy.

**Conclusion:**

We incorporated an educational program on high-value cost-conscious care in the training of ICU physicians. Based on our vignette study, we conclude that the improvement of knowledge of costs and prognosis after this program was limited.

## Background

With a growing elderly population, technical innovations, and new treatment possibilities, healthcare is facing new challenges in high quality but cost- effective care. The Netherlands has one of the best healthcare systems in the world, however, maintaining this position is expensive, accounting for about 10% of the Dutch Gross National Product in 2017 [1, 2]. Intensive Care Units (ICU) offer opportunities for extended and long-lasting support of vital functions and organs, however the costs are high. Wammes et al. showed in 2017 that 9,1% of the total costs of Dutch hospital health care is spend on ICU care [3]. The difference in cost between ICU and non-ICU care is at least $1000 per day [4]. The past decades this difference has quadrupled [5]. However, not all ICU care results automatically in favorable outcomes [6, 7].

Careful decisions on the use of critical care can only be made when physicians are fully acquainted with the potential clinical outcomes as well as the costs of ICU care. However, the level of awareness regarding the costs of common prescription drugs (especially high cost medication), and commonly used materials in the ICU is often lacking, as described by Hernu et al. [8] Moreover, ICU physicians generally overestimate the long-term survival and quality of life of ICU survivors [9]. This lack of knowledge may be attributed to the lack of training about cost awareness and outcome of ICU treatment in the current postgraduate medical training program [10].

The Awareness project was launched in the Netherlands in 2017–2018 by the Federation of Medical Specialists and Maastricht University, to try to incorporate awareness on value and expenditure in addition to the CANMED competencies in all post graduate medical training programs. Its goals were to preserve high quality care, while at the same time creating more awareness regarding the appropriateness of care and raise cost-consciousness [11]. This is also called High Value Cost-Conscious care [12]. To launch the Awareness project, all regional medical education programs were invited to apply for projects on this matter. The transmission of knowledge, reflective practice, and a supportive environment are important elements in training physiscians [13].

An educational program for ICU residents and fellows enhancing knowledge on outcome and costs of ICU care was carried out at the Intensive Care Unit of the Amsterdam University Medical Centre. The present study aims to assess the effect of this educational program on the knowledge of treatment outcomes and associated costs of ICU support.

## Methods

### Design

In this cohort study we used a factorial survey design in which participants were asked to evaluate clinical vignettes. Hereby importance of factors influencing decision making could be assessed. A vignette is a brief, written case history of a fictitious patient that is based on a realistic clinical situation. In the vignettes the factors of interest (in our study possible predictors of ICU outcome) were varied between the different vignettes, in which each combination was unique. We made combinations between categories of predictors, in which only one factor changed between each vignette. These vignettes were presented to the participant, residents and fellows in our ICU, in an online questionnaire using the Survey Monkey website [14]. Formal approval of the institutional ethics committee was not requested, participants were informed about the anonymized use of the test results and participation was voluntary. Due to the nature of the project no sample size calculations were made.

The online questionnaire consisted of 23 clinical vignettes, in which four common ICU admission diagnoses were presented; community acquired pneumonia (CAP), pancreatitis, acute respiratory distress syndrome (ARDS) and cardiac arrest. Admission diagnoses were discussed between authors and chosen for because CAP and OHCA are among the five most common admission diagnosis in the Netherlands in 2018 [15] and our hospital is a tertiary referral centre for pancreatitis and ARDS. Furthermore, all these diagnoses have well known predictors for outcome [16, 17]. The literature was studied for patient factors known to have impact on patient outcome in ICU for these conditions. With this information an expert panel of ICU physicians decided to add the following factors to the vignettes: age, body mass index (BMI), acute kidney injury (AKI) and presence of haematological malignancy for all diseases, and presence of chronic obstructive pulmonary disease (COPD) and severity of pneumonia measured by CURB-65 score for pneumonia, presence of chronic liver disease and severity of pancreatitis measured by Ranson score for pancreatitis, severity of ARDS and type of ARDS (distinguishing primary and secondary ARDS) for ARDS and for the cardiac arrest cases first rhythm (shockable versus non-shockable), location of arrest (in hospital versus out of hospital) and delay until start CPR [16–23]. All community acquired pneumonia cases were classified as severe, according to the CURB-65 score [18]. In cases for ARDS and cardiac arrest all patient had a BMI of 20–25 and an age of 60–80 years old. A complete overview of all factors involved is shown in Table 1.

**Table 1:** Variables in the clinical vignettes

All cases	Presence of haematological malignancy	Yes/no
	Acute kidney failure	Yes/no
	Age	< 60 years/ 60–80 years/ > 80 years
	BMI	< 20 / 20–25 / > 25
Community acquired pneumonia	Presence of COPD	Absent / mild / severe
	Severity of Pneumonia (measured CURB −65 score)	CURB-65 score 0–1 / 2 / 3–5
Acute Pancreatitis	Presence of chronic liver disease	Absent/ Liver cirrhosis Child Pugh A/ B/ C
	Severity of pancreatitis, measured by Ranson score	Ranson Score 3–4/ 5–6/ 7–8
ARDS	Type of ARDS	Primary/ Secondary
	Severity of ARDS	None/ Mild/ Moderate/ Severe
Cardiac arrest	First rhythm	Shockable (VF/ VT)/ Non-shockable (PEA/ Asystole)
	Location of arrest	Out of hospital cardiac arrest/ In hospital cardiac arrest
	Delay until start CPR	< 3 min/ > 3 min

*BMI* body mass index, *COPD*chronic obstructive pulmonary disease, *CURB-65 score* confusion, urea, respiratory rate and blood pressure- score, *ARDS* acute respiratory distress syndrome, *VF* ventricular fibrillation, *VT* ventricular tachycardia, *PEA* pulseless electric activity, *CPR* cardiopulmonary resuscitation

With the complete set of factors, 324 vignettes for community acquired pneumonia (2x2x3x3x3x3), 432 vignettes for pancreatitis (2x2x3x3x4x3), 288 vignettes for ARDS (2x2x3x3x2x4) and 288 vignettes for cardiac arrest (2x2x3x3x2x2x2) were created. Completing all 1332 vignettes would be too time-consuming for participants. In each vignette only one factor changed to the following vignette. A total of 23 clinically vignettes (6 pneumonia, 7 pancreatitis, 4 ARDS and 6 cardiac arrest) were selected by a team of experts/ICU clinicians based on their realistic scenarios. The vignettes were written by one of the investigators (LW) and discussed by the other investigators (MM and CB) on clinical accuracy and realism. The complete questionnaire can be found in Supplement 1.

For the vignettes on CAP and pancreatitis, participants were asked to choose between admission or no admission to the ICU, taking into account chances of survival and treatment. For all vignettes with ARDS or cardiac arrest participants were asked to estimate mortality during first 30 days of ICU stay. The options were < 40% mortality, 40–80% mortality or > 80% mortality.

Also, five questions were added on the cost of five products, which are frequently used in the ICU (arterial blood gas analysis, Computed Tomography (CT) scan of the head, platelet transfusion (one unit), immune-modulating enteral feeding, forced-air heating blanket). Each question was multiple choice and respondents were asked to pick the right price for the product.

Demographics of the participants were collected at the start of the questionnaire. Recorded variables included working experience in ICU care, previous experience in residency or fellowship and primary postgraduate medical education programs. The first questionnaire was carried out in January 2018, the second, after the educational intervention, in April 2018. The questionnaire was carried out in January 2018, and again, after the educational intervention, in April 2018.

### Setting

This study was conducted at the Intensive care department of the Amsterdam University Medical Center, location Academic Medical Center, at the University of Amsterdam. The department is a 34-bed mixed medical-surgical ICU, where residents of various postgraduate medical education programs are trained (e.g. internal medicine, anaesthesiology, surgery, neurosurgery, cardiology, emergency medicine). In addition, each year 7 fellows are trained to become an intensivist as a subspecialty of their training in anaesthesiology, internal medicine, cardiology or neurology.

### Participants

The study population consisted of ICU residents with medical training in anaesthesiology, internal medicine, emergency medicine, neurosurgery, cardiothoracic surgery or general surgery, and fellows in Intensive Care Medicine, with a medical specialization in internal medicine, anaesthesiology or neurology. All physicians were employed on the ICU of the Amsterdam Medical Centre, a tertiary clinic, during the study period.

### Intervention

Between January and April 2018 an educational program on outcome and cost of ICU treatment was implemented in the regular educational program. This program consisted of flipped classroom sessions on outcomes of patients admitted to the ICU with a certain illness, comorbidity or patient characteristic; lectures on costs of ICU care and cost reduction and organization of ICU and a weekly quiz [24]. The flipped classroom sessions focused on COPD, haematological malignancies, pancreatitis, community acquired pneumonia, cardiac arrest, age and BMI. In total eight different flipped classroom sessions were organized. The classical lectures focused on organization of ICU care, assessing the outcomes of ICU care, cost reduction on laboratory measurements and financing ICU care. Since all of our physicians work on irregular shifts all lectures were held twice and handouts of the lectures and acquired information of the flipped classroom sessions were shared between all physicians.

The weekly quiz with a fictional but realistic case and a question on the actual costs of a certain investigation, medication or material was sent to all participants every Monday. The aim was to draw more attention to the project and stimulate discussion among participants.

### Potential effect modifiers, confounders and bias

Potential modifiers of the measured effect of the intervention are previous experience in ICU care, outcome of ICU care, cost and already gathered knowledge on high-value cost-conscious care during general medical training or residency. For this reason, we included all residents and fellows employed at our ICU during the intervention and asked about their level of experience in ICU care. There was risk of inclusion bias, since participation was voluntary and this could select only participants interested in the subject of the study. To prevent selection bias we briefed all residents and fellows about the study and potential of high-value cost-conscious care education to gain interest in the study. We tried to minimize loss of follow up by asking participants to complete the final questionnaire on several occasions.

### Statistical analysis

Descriptive statistics were used to describe the characteristics of the participating residents. We used logistic regression to determine the first outcome, relative importance of the factors for ICU admission, in which ICU admission was set as determinant and the factors as independent variables. Data are shown as odds ratios (OR) with 95% confidence interval.

For the second outcome, the importance of the factors for the estimate of mortality, we used univariate multi-nominal regressing, with percentage of survival as determinant and the factors as independent variables. Data are shown as odds ratios (OR) with 95% confidence interval.

The third outcome, the estimation of costs of products used regularly in ICU, was noted a percentage different from the true cost. Results are shown as means with standard error of the mean (SEM). A students unpaired t-test was performed to compare answers before and after education.

IBM Spss version 25 was used for the statistical analyses and a p value of < 0.05 was considered statistically significant.

## Results

### Participants

All residents and fellows employed at our ICU during the intervention were included in the study. The pre-education online questionnaire was completed by 32 participants (100% of those who attended) and the post-education questionnaire by 27 participants (84%). Loss to follow up of 5 participants was due to termination of the internship at ICU, after which they changed hospitals. There was no missing data. All the participants answered all the questions in the questionnaire. The majority of respondents had a background in anaesthesiology (56% and respectively 56%) and Internal medicine (25% and respectively 26%). Nine respondents had over 1 year of experience in Intensive Care, while four had no experience at all (Table 2).

**Table 2:** Demographics of participants of the online questionnaire

	Pre-education *n* = 32 (100%)	Post-education *n* = 27 (84%)
Background		
Anaesthesiology	18 (56%)	15 (56%)
Internal Medicine	8 (25%)	7 (26%)
Emergency Medicine	1 (3%)	1 (4%)
Cardiology	2 (6%)	2 (7%)
Neurology	1 (3%)	1 (4%)
Cardiothoracic surgery	1 (3%)	1 (4%)
Neurosurgery	1 (3%)	0 (0%)
Year of residency		
1st year of residency	0 (0%)	1 (4%)
2nd year of residency	10 (31%)	6 (22%)
3rd year of residency	14 (44%)	13 (48%)
Fellowship ICU	8 (25%)	7 (26%)
Experience in ICU care		
None	4 (13%)	3 (11%)
0–3 months	6 (19%)	2 (7%)
3–6 months	8 (25%)	8 (30%)
6–12 months	5 (16%)	5 (19%)
More than 1 year	9 (28%)	9 (33%)

*ICU* Intensive Care Unit

### Predictive factors for ICU admission and mortality

Results are shown in Table 3. Patients with a haematological malignancy (OR 2.084 (1.257–3.454), BMI > 25 (OR 2.931(1.008–8.256) were more likely to be admitted to the ICU prior to the educational program. Patients with a pancreatitis with a Ranson score of 5–6 were less likely to be admitted to the ICU (OR 0.054 (0.019–0.156), compared to pancreatitis with Ranson score 3–4. After the educational program the effects of factors haematological malignancy and BMI > 25 were attenuated. The effect of the Ranson score 5–6 remained after education (OR 0.129 (0.050–0.034).

**Table 3:** Odds ratios for the admission of the patient to the ICU

Factor	Pre-education		Post-education	
	OR (95% CI)	*p*-value	OR (95% CI)	*p*-value
Presence of haematological malignancy	2.084 (1.257–3.454)	0.004	1.021 (0.582–1.789)	0.943
Acute kidney failure	0.694 (0.427–1.127)	0.140	0.803 (0.465–1.388)	0.433
Age				
< 60 years	reference		reference	
60–80 years	0.576 (0.231–1.438)	0.237	0.584 (0.214–1.590)	0.292
> 80 years	0.444 (0.142–1.394)	0.164	0.543 (0.153–1.931)	0.346
BMI > 25	2.931 (1.008–8.526)	0.048	3.228 (0.950–10.969)	0.060
Presence of COPD	0.890 (0.560–1.414)	0.622	0.866 (0.538–1.393)	0.553
Severity of Pancreatitis, measured by Ranson score [12]				
3–4	Reference			
5–6	0.054 (0.019–0.156)	0.000	0.129 (0.050–0.334)	0.000
7–8	0.664 (0.353–1.247)	0.203	0.626 (0.289–1.354)	0.234
Previous history of liver disease	0.633 (0.782–1.161)	0.633	1.000 (0.503–1.987)	1.000

The OR represents the average odd that the patient is likely to be not admitted to the ICU

*OR* odds ratio, *CI* confidence interval, *BMI* body mass index, *COPD* chronic obstructive pulmonary disease

Outcomes of the multinomial regression for factors potentially influencing the estimate of mortality are shown in Table 4. Prior to the educational program location of cardiac arrest (in- vs. out of hospital cardiac arrest) was perceived to be associated with mortality (estimated mortality > 80%: OR 9.274 (1.2–71.697). After the educational program only the presence of a haematological malignancy was considered to be an important factor in the estimation of mortality (estimated mortality 40–80%: OR 4.320 (2.089–8933) and estimated mortality > 80%: OR 3.124 (1.497–6.519).

**Table 4:** Factors of importance in the estimation of mortality in ARDS and cardiac arrest

Factor	Pre- education				Post- education			
	Mortality 40–80%		Mortality > 80%		Mortality 40–80%		Mortality > 80%	
	OR (CI)	p	OR (CI)	p	OR (CI)	p	OR (CI)	p
Presence of haematological malignancy	0.188 (0.042–0.847)	0.029	0.161 (0.036–0.720)	0.017	4.320 (2.089–8.933)	0.000	3.124 (1.497–6.519)	0.002
Acute kidney failure	0.056 (0.007–0.426)	0.005	0.023 (0.003–0.169)	0.000	0.79 (0.010–0.604)	0.014	0.024 (0.003–0.177)	0.000
Location of cardiac arrest	6.740 (0.846–53.708)	0.072	9.274 (1.2–71.697)	0.033	NA	NA	0.406 (0.168–0.981)	0.045
Delay until start CPR	0.045 (0.010–0.199)	0.00	0.021 (0.005–0.94)	0.000	0.038 (0.005–0.294)	0.02	0.013 (0.002–0.100)	0.000
First heart rhythm in cardiac arrest	0.610 (0.283–1.314)	0.207	0.631 (0.297–1.343)	0.232	0.541 (0.269–1.555)	0.330	0.541 (0.225–1.297)	0.168
Severity of ARDS	0.065 (0.018–0.236)	0.000	0.012 (0.003–0.055)	0.000	0.056 (0.007–0.443)	0.006	NA	NA

*OR* odds ratio, *CI* confidence interval 95%, *CPR* cardiopulmonary resuscitation, *ARDS* acute respiratory distress syndrome, *OHCA* out of hospital cardiac arrest

### Cost of ICU care

**Fig. 1:**
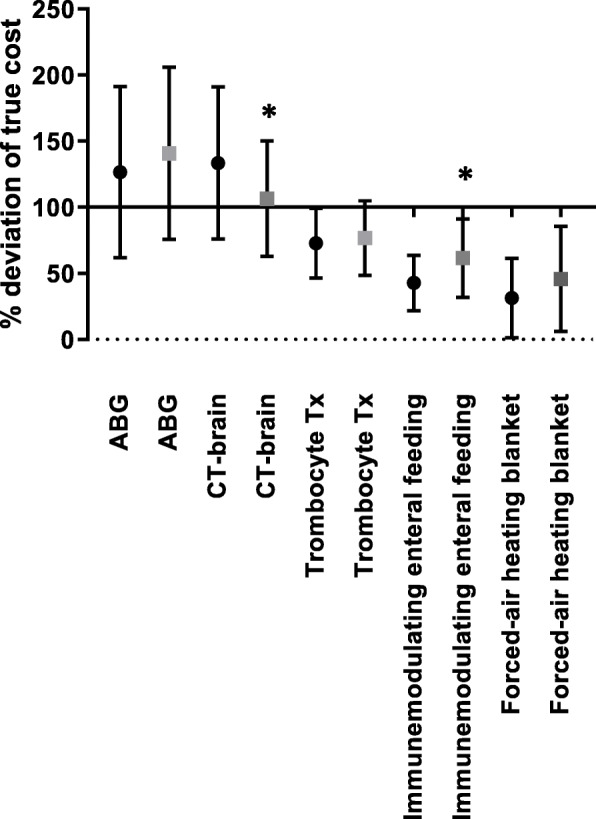
Percentage deviation of true cost of five different ICU products or diagnostic procedures. Dots are pre-education, squares are post-education. Results are shown in means ±SEM. X-axis at 100% is the true cost of the product or procedure. All values close to the X-axis are approximating true product cost. * is statistically significant difference (*P* < 0.05) between pre- and post-education. Abbreviations: ABG = arterial blood gas analysis, CT = computed tomography, Tx = transfusion

All participants filled out all the questions on costs of ICU products. After the educational program the questions on costs showed reduced deviation of the true costs, except arterial blood gas analysis (Fig. 1). The deviation of cost for this item is higher, before the education participants underestimated the costs and after education the costs were overestimated to a greater extent. Answers on CT-brain (pre-education mean percentage 113.5% ± 10.8, post-education 106.5% ±8.4, *p* = 0.049) and immune-modulating enteral food (pre-education 42.75% ±3.7, post-education 61.52% ±5.7, *p* = 0.0063) both significantly showed reduced deviation in regard to the true product cost after education.

## Discussion

We implemented an educational program for residents and fellows on the outcome of ICU care in a tertiary academic ICU in the Netherlands. The program was designed to enhance knowledge about outcome and mortality of the most common ICU diagnoses and comorbidities. The program consisted of flipped class room sessions on outcome of care, classical lectures on benchmarking ICUs, organization of ICU and costs of ICU care, and a weekly quiz on costs of products often used in our ICU.

We used a clinical vignette study to evaluate the learning effect of our educational program. A vignette study can show which factors are pivotal in the decision whether to admit a certain patient to the ICU.

### Interpretation

Participating physicians showed to make different decisions pre- versus post-education. A BMI over 25 and presence of a haematological malignancy were considered an important factor for ICU admission pre-education, however this effect was not measured after the educational program. In the teaching program residents learned that a BMI > 25 can be protective in certain situations [21–23]. In addition, they also learned that the presence of any haemato-oncological disease results in high morbidity and mortality, and therefore reduces a positive effect of ICU care. The net effect of this increased awareness resulted in a lower odds ratio when considering a haematological-oncological disease as a factor for admittance after the educational program (Table 3) [25–27]. However, increased awareness on factors that influence the outcome of ICU admission was limited, and did not improve after the educational program. The latter was shown by the pancreatitis vignette where patients with more severe disease (expressed as Ranson’s criteria) where less likely to be admitted to the ICU. The educational program did lead to a shift in knowledge on predictive factors in case of mortality in ARDS or cardiac arrest. While pre-education, the location of the cardiac arrest (in hospital versus out of hospital) was marked as the most important factor, post education the most important factor shifted to the presence of a haematological malignancy.

The current project was carried out as a part of the Dutch Awareness project, which is a successor to the Choosing Wisely campaign, launched by the American Boards of Internal Medicine (ABIM) Foundation in 2012 [28]. The influence of the latter was evaluated by Rosenberg in 2015, who studied the volume of change of seven low-value services mentioned in the Choosing Wisely campaign [29]. Low-value services are considered avoidable treatments and tests that are unnecessary or harmful. In only two recommendations a small decrease could be found (imaging for headache and cardiac imaging without history), showing the difficulty of implementing high-value cost-conscious care. Despite the short duration of our study and its small sample size, our study shows the same difficulty in implementing high-value cost-conscious care. In order to achieve a health system in which high-value cost-conscious care becomes the default mindset, a need for cultural change and involvement of the medical societies may be required as described by Kerr in 2017 [30]. To deliver high-value cost-consciousness care, the theme should be implemented in (post-graduate) medical training programs and incorporated in daily work regimes in order to reach maximum effect..

### Limitations

There are several limitations to our study. For feasibility reasons the number of clinical vignettes was set to 23. In creating the vignettes only one of the factors could change between each case; this limited the number of factors used in our vignettes. Also, the range in answers was smaller than expected; this resulted in insufficient data to perform statistical analysis for some factors or categories. Future studies should therefore include a larger number of participants, so more vignettes can be developed and divided between participants.

The residents completing the questionnaire probably also experienced a learning effect from their ICU internship next to the supplied additional educational program. We cannot differentiate between the acquired knowledge from the educational program and the effect of their day to day job (being on call, treating patients etc.). Also, while this program was scheduled during normal working hours, some residents could not attend all classes. To this extent, all lectures were available online to the whole group, also those who did not attend the class in person. However, we do not have any data on which lectures were reviewed by those who did not attend. Hence, we were not able to relate the level of participation to the eventual learning effect of the program. The decision to admit a patient to ICU is often based on clinical judgement, combined with knowledge on predictors of outcome. Moreover, overuse and inappropriate use of ICU care should be limited as much as possible. Participants were asked to choose between ICU- admission or withholding admission in some of the vignettes, taking account the expected benefit of ICU care. This might be a hard choice. The consensus between ICU physicians whether a patient will benefit from ICU admission is poor, as was shown by Valley et al. [31] At most, only 69% of physicians agreed about the extent to which a patient would benefit from ICU care. The lack of overall agreement between ICU physicians is worrisome and may also lead to disproportionate health care costs. It also indicates that the question whether to admit a patient to ICU, aiming benefit for the patient, is not an easy one to answer and might be adjusted in future studies.

## Conclusion

The basic knowledge on ICU mortality and contributing factors is limited in residents and fellows at our ICU and there is a need to improve this knowledge to improve cost-conscious decision making in an era of rising health care costs. Our study shows the possible implications of an educational program focusing on knowledge about ICU admission. Based on our vignette study, we conclude that the improvement of knowledge of costs and prognosis after this program was limited. With this information we would like to improve our educational program and assess the outcomes within a larger sample size and over a longer period.

## Supplementary information

**Additional file 1.** Translation complete vignette questionnaire.

## Data Availability

All data is anonymised. The data used and/ or analyzed can be available by the corresponding author on request.

## Abbreviations

ABIM: American Board of Internal Medicine
ARDS: Acute respiratory distress syndrome
AKI: Acute kidney injury
BMI: Body mass index
CAP: Community acquired pneumonia
COPD: Chronic obstructive pulmonary disease
CPR: Cardiopulmonary resuscitation
CT: Computed tomography
ICU: Intensive Care Unit
OR: Odds ratio
SEM: Standard error of the mean
