# Supplementary material for: Program on high value cost-conscious education in intensive care: Educational program on prediction of outcome and cost awareness on Intensive Care admission
Source: BMC Med Educ. 2020 Jun 8;20:186. doi: 10.1186/s12909-020-02100-w (PMC7282117; doi:10.1186/s12909-020-02100-w)
Supplement: Supplementary file 1 — Additional file 1. Translation complete vignette questionnaire. [file 12909_2020_2100_MOESM1_ESM.pdf]

Additional files

Supplement 1

File name: Translation complete vignette questionnaire

This supplement is the complete translation of the questionnaire we offered our participant at the start of the study and after our intervention. The questionnaire consists of 4 parts:

- Part 1: Demographic questions
- Part 2: Fictitious but realistic cases.
  - The participants has to answer the question
    - Would you admit the patient to your ICU?
    - Answer possibilities are: Yes or No
- Part 3: Fictitious but realistic cases.
  - The participant has to answer the question
    - How do you estimate the mortality of this patient?
    - Answer possibilities are mortality <40%, mortality 40-80% and mortality > 80%
- Part 4: Estimation of costs of products used daily in the ICU.

Page 1

Q1. Are you:

A. Resident 1st year of training
B. Resident 2nd year of training
C. Resident 3rd year of training
D. Fellow Intensive Care

Q2. What is your primary medical training program?

A. Internal medicine
B. Anaesthesiology
C. Emergency Medicine
D. Cardiology
E. Neurology
F. Cardiothoracic surgery
G. Neurosurgery

Q3. What is your experience level in Intensive Care medicine, measured in time, at the start of this project?

A. None
B. 0-3 months
C. 3-6 months
D. 6-12 months
E. > 1 year

Page 2

You will be given several fictitious, but realistic cases. Please answer whether you would admit the patient to the ICU.

Q4. A 58-year-old woman without medical history was evaluated at the emergency department with acute onset dyspnoea. The most likely cause is community acquired pneumonia. Her BMI is 23. She has a respiratory rate of 23/min, she has a reduced level of consciousness and her blood pressure is 80/40mmHg. Would you admit this patient to your ICU?

A. Yes
B. No

Q5. A 58-year-old woman with a medical history of chronic obstructive pulmonary disease (COPD) is evaluated at the emergency department with acute onset dyspnoea. The most likely cause is community acquired pneumonia. Her BMI is 23. She has a respiratory rate of 23/min, she has a reduced level of consciousness and her blood pressure is 80/40mmHg. She has been admitted in the hospital due to acute COPD exacerbation 3 times in the past year. One time she has been supported by mechanical invasive ventilation. The bicarbonate level in her blood gas analysis is 38 mmol/L. This patient continues smoking and she uses oxygen at home, for 2L/min. Would you admit this patient to your ICU?

A. Yes
B. No

Q6. A 58-year-old woman is evaluated at the emergency department with acute onset dyspnoea. She has a known history of acute myeloid leukaemia for which she was treated by an allogenic stem cell transplantation, but recently a relapse was diagnosed and she started on chemotherapy. The most likely cause of her dyspnoea is community acquired pneumonia. Her BMI is 23. She has a respiratory rate of 23/min, she has a reduced level of consciousness and her blood pressure is 80/40mmHg. Would you admit this patient to your ICU?

A. Yes
B. No

Q7. A 58-year-old woman is evaluated at the emergency department with acute onset dyspnoea. She has a known history of acute myeloid leukaemia for which she was treated by an allogenic stem cell transplantation, but recently a relapse was diagnosed and she started on chemotherapy. The most likely cause of her dyspnoea is community acquired pneumonia. Her BMI is 23. She has a respiratory rate of 23/min, she has a reduced level of consciousness and her blood pressure is 80/40mmHg. Her creatinine level was 180umol/L, which has been 58umol/L at her last clinical check-up. Would you admit this patient to your ICU?

A. Yes
B. No

Q8. A 76-year-old woman is evaluated at the emergency department with acute onset dyspnoea. She has a known history of acute myeloid leukaemia for which she was treated by an allogenic stem cell transplantation, but recently a relapse was diagnosed and she started on chemotherapy. The most likely cause of her dyspnoea is community acquired pneumonia. Her BMI is 23. She has a respiratory rate of 23/min, she has a reduced level of consciousness and her blood pressure is 80/40mmHg. Her creatinine level was 180umol/L, which has been 58umol/L at her last clinical check-up. Would you admit this patient to your ICU?

A. Yes
B. No

Q9. A 58-year-old woman without medical history is evaluated in the emergency department with acute onset dyspnoea. The most likely cause is community acquired pneumonia. Her BMI is 43. She has a respiratory rate of 23/min, she has a reduced level of consciousness and her blood pressure is 80/40mmHg. Would you admit this patient to your ICU?

A. Yes
B. No

Page 3

You will be given several fictitious, but realistic cases. Please answer whether you would admit the patient to the ICU.

Q10. A 43-year-old man is evaluated in the emergency department with abdominal pain, most likely caused by pancreatitis. His BMI is 23 and he has no medical history. His leukocytes are 22x 10^9/L, ASAT is 351U/L, creatinine is 89umol/L and glucose is 12 mmol/L. His vital parameters are stable, but he makes a sick impression on you. Would you admit this patient to your ICU?

A. Yes
B. No

Q11. A 43-year-old man is evaluated in the emergency department with abdominal pain, most likely caused by pancreatitis. His BMI is 23 and he has no medical history. His leukocytes are 22x 10^9/L, ASAT is 351U/L, LDH is 466U/L and glucose is 12 mmol/L. He received four liters of fluid on presentation and keeps in need of fluid resuscitation. An arterial blood gas analysis shows a paO2 of 7.6kPa, base excess -8. Would you admit this patient to your ICU?

A. Yes
B. No

Q12. A 82-year-old man with no known medical history is evaluated in the emergency department with abdominal pain, most likely to be caused by pancreatitis. His BMI is 23. His leukocytes are 22x 10^9/L, ASAT is 351U/L, LDH is 466U/L and glucose is 12 mmol/L. He received four liters of fluid on presentation and keeps in need of fluid resuscitation. An arterial blood gas analysis shows a paO2 of 7.6kPa, base excess -8. Would you admit this patient to your ICU?

A. Yes
B. No

Q13. A 43-year-old man with no known medical history is evaluated in the emergency department with abdominal pain, most likely caused by pancreatitis. His BMI is 23. His leukocytes are 22x 10^9/L, ASAT is 351U/L, LDH is 466U/L and glucose is 12 mmol/L. His creatinine level is 230umol/L. Would you admit this patient to your ICU?

A. Yes
B. No

Q14. A 43-year-old man is evaluated in the emergency department with abdominal pain, most likely caused by pancreatitis. His BMI is 23 and he has an alcoholic liver cirrhosis (Child Pugh C). His leukocytes are 22x 10^9/L, ASAT is 351U/L, LDH is 466U/L and glucose is 12 mmol/L. Since entering the emergency department, he has received over 4 liters of fluid resuscitation. His

base excess is -8 and the PaO2 is 6.8kPa in his arterial blood gas analysis. Would you admit this patient to your ICU?

A. Yes
B. No

Q15. A 43-year-old man is evaluated in the emergency department with abdominal pain, most likely caused by pancreatitis. His BMI is 23 and he has an alcoholic liver cirrhosis (Child Pugh C). His leukocytes are 22x 10^9/L, ASAT is 351U/L, LDH is 466U/L and glucose is 12 mmol/L. Since entering the emergency department, he has received over 4 liters of fluid resuscitation and he remains dependent of ongoing fluid resuscitation. His base excess is -8 and the PaO2 is 7.6 kPa in his arterial blood gas analysis. Would you admit this patient to your ICU?

A. Yes
B. No

Q16. A 43-yearold man is evaluated in the emergency department with abdominal pain, most likely caused by pancreatitis. His BMI is 23 and he has an acute myeloid leukaemia, which was treated recently by allogenic stem cell transplantation. His leukocytes are 22x 10^9/L, ASAT is 351U/L, LDH is 466U/L and glucose is 12 mmol/L. Since entering the emergency department, he has received over 4 liters of fluid resuscitation and he remains dependent of ongoing fluid resuscitation. Would you admit this patient to your ICU?

A. Yes
B. No

Page 4

You will be given several fictitious, but realistic cases. You are about to inform the family of the patient who was just admitted to your Intensive Care Unit. Please answer what your estimated mortality is of this patient.

Q17. A 76-year-old man with a normal BMI is admitted to the ICU with blunt thoracic trauma (multiple rib fractures and right sided lung contusion). His infectious parameters decline after 5 days and his hemodynamic support is no longer necessary. He develops acute respiratory failure and needs to be supported by mechanical ventilation. His ventilatory settings increase and you suspect him of ARDS, according to the Berlin criteria. The thoracic X-ray shows bilateral infiltration of the lungs and there is no cardiac failure. The ventilatory peak pressure is 32cmH2O and P/F ratio is 178mmHg and 24kPa. This is mono-organ failure. How do you estimate the mortality of this patient?

A. Mortality < 40%
B. Mortality 40-80%
C. Mortality > 80%

Q18. A 76-year-old man with a normal BMI is admitted to the ICU with perforated diverticulitis. He is clinically improving after 3 days, but then suddenly develops acute respiratory failure and needs to be supported by mechanical ventilation. His ventilatory settings increase and you suspect him of ARDS, according to the Berlin criteria. The thoracic X-ray shows bilateral infiltration of the lungs and there is no cardiac failure. The ventilatory peak pressure is 32cmH2O with a respiratory rate of 30/min. The P/F ratio is 178mmHg and 24kPa. This is mono-organ failure. How do you estimate the mortality of this patient?

A. Mortality < 40%
B. Mortality 40-80%
C. Mortality > 80%

Q19. A 76-year-old man with a normal BMI is admitted to the ICU with blunt thoracic trauma (multiple rib fractures and right sided lung contusion). His infectious parameters decline after 5 days and his hemodynamic support is no longer necessary. He develops acute respiratory failure and needs to be supported by mechanical ventilation. His ventilatory settings increase and you suspect him of ARDS, according to the Berlin criteria. The thoracic X-ray shows bilateral infiltration of the lungs and there is no cardiac failure. The ventilatory peak pressure is 38cmH2O with a respiratory rate of 35/min. The P/F ratio is 80mmHg and 10,6kPa. You have started diuretics, but without result yet. You decide to place the patient in prone position. This is mono-organ failure. How do you estimate the mortality of this patient?

A. Mortality < 40%
B. Mortality 40-80%
C. Mortality > 80%

Q20. A 76-year-old man with a normal BMI is admitted to the ICU with blunt thoracic trauma (multiple rib fractures and right sided lung contusion). His infectious parameters decline after 5 days and his hemodynamic support is no longer necessary. He develops acute respiratory failure and needs to be supported by mechanical ventilation. His ventilatory settings increase and you suspect him of ARDS, according to the Berlin criteria. The thoracic X-ray shows bilateral infiltration of the lungs and there is no cardiac failure. The ventilatory peak pressure is 38cmH2O with a respiratory rate of 35/min. This results in a pH 7.26. The PF ratio is 80mmHg and 10,6kPa. You have started diuretics, but without result yet. Creatinine levels have risen to 380umol/L, potassium 5.8 mmol/L. You decide to place the patient in prone position after inserting a CRRT (continuous renal replacement therapy)-line. How do you estimate the mortality of this patient?

A. Mortality < 40%
B. Mortality 40-80%
C. Mortality > 80%

Page 5

You will be given several fictitious, but realistic cases. You are about to inform the family of the patient who was just admitted to your Intensive Care Unit. Please answer what your estimated mortality is of this patient.

Q21. A 74-year-old man with no medical history and a normal posture has a cardiac arrest at his home. The delay until start CPR is minimal, because his son-in-law started CPR immediately. The arrest was witnessed. The first rhythm after arrival of the paramedics was pulseless electric activity (PEA). He develops return of spontaneous circulation (ROSC) after 3 doses of adrenaline. During intubation food is taken out of the trachea. He is admitted to the Intensive Care for Targeted Temperature Management(TTM). What do you tell his relatives about his estimated mortality?

A. Mortality < 40%
B. Mortality 40-80%
C. Mortality > 80%

Q22. A 74-year-old man with no medical history and a normal posture has a cardiac arrest at his home. The delay until start CPR is minimal, because his son-in-law started CPR immediately. The arrest was witnessed. The first rhythm after arrival of the paramedics was ventricular fibrillation. He was defibrillated three times and develops ROSC. He has electrocardiogram (ECG)-abnormalities suspected for myocardial infarction and after percutaneous coronary intervention (PCI) of his coronaries, he is admitted to the Intensive Care for Targeted Temperature Management. What do you tell his relatives about his estimated mortality?

A. Mortality < 40%
B. Mortality 40-80%
C. Mortality > 80%

Q23. A 74-year-old man with no medical history and a normal posture has a cardiac arrest at his home. He was found by his wife, who ran to the neighbours to get help. The firefighters started CPR and attached the AED. The first rhythm after arrival of the paramedics was ventricular fibrillation. He was defibrillated five times and develops ROSC. Delay before start CPR was estimated to be at least 10 minutes. He has ECG abnormalities suspected for myocardial infarction and after PCI of his coronaries, he is admitted to the Intensive Care for Targeted Temperature Management. What do you tell his relatives about his estimated mortality?

A. Mortality < 40%
B. Mortality 40-80%
C. Mortality > 80%

Q24. A 74-year-old man with a normal posture has a cardiac arrest on the ward. He was admitted in a single room with a pneumonia and was treated with antibiotics for 3 days before this event. The ward nurses noted in his file that he has had elevated respiratory rates and was

not feeling well. The nurses found him unresponsive in his bed and started CPR. The first rhythm seen was a PEA. After 20 minutes he develops ROSC. The most likely cause of the cardiac arrest is hypoxemia due to mucus plugging. The patient is admitted to the Intensive Care for further treatment. What do you tell his relatives about his estimated mortality?

A. Mortality < 40%
B. Mortality 40-80%
C. Mortality > 80%

Q25. A 74-year-old-man with a normal posture has a cardiac arrest at home. The delay until start CPR is minimal because his son in law started CPR immediately. The arrest was witnessed. The first rhythm after arrival of the paramedics was ventricular fibrillation. He was defibrillated five times and develops ROSC. He has no ECG abnormalities and after excluding other causes, the cardiac arrest is most likely to be caused by previous myocardial damage. The patient is known by the haematologist, who treated him for a multiple myeloma with allogenic stem cell transplantation. Because of a relapse he is treated with chemotherapy. The patient is admitted to the Intensive Care for Targeted Temperature Management. What do you tell his relatives about his estimated mortality?

A. Mortality < 40%
B. Mortality 40-80%
C. Mortality > 80%

Page 6

Please answer the next questions and estimate the cost of various commonly used ICU products

Q26. What does it cost to perform an arterial blood gas analysis?

A. €2,14
B. €4,14
C. €10,28
D. €8,28

Q27. What does a CT-cerebrum without contrast cost?

A. €54,00
B. €78,00
C. €125,00
D. €254,00

Q28. What are the costs of one thrombocyte transfusion?

A. €556,15
B. €203,19
C. €185,00
D. €468,89

Q29. What is the cost of 500mL of immunomodulating enteral feeding (Impact)

A. €52,12
B. €92,12
C. €152,12
D. €192,12

Q30. How much does one forced air warming blanket cost (Bairhugger blanket)?

A. €8,14
B. €80,14
C. €180,14
D. €280,14
